# Association of Birth Year of Pregnant Individuals With Trends in Hypertensive Disorders of Pregnancy in the United States, 1995-2019

**DOI:** 10.1001/jamanetworkopen.2022.28093

**Published:** 2022-08-24

**Authors:** Natalie A. Cameron, Lucia C. Petito, Nilay S. Shah, Amanda M. Perak, Janet M. Catov, Natalie A. Bello, Simon Capewell, Martin O’Flaherty, Donald M. Lloyd-Jones, Philip Greenland, William A. Grobman, Sadiya S. Khan

**Affiliations:** 1 Division of Internal Medicine and Geriatrics, Department of Medicine, Northwestern University Feinberg School of Medicine, Chicago, Illinois; 2 Department of Preventive Medicine, Northwestern University Feinberg School of Medicine, Chicago, Illinois; 3 Division of Cardiology, Department of Medicine, Northwestern University Feinberg School of Medicine, Chicago, Illinois; 4 Division of Cardiology, Department of Pediatrics, Ann & Robert H. Lurie Children’s Hospital of Chicago, Chicago, Illinois; 5 Stanley Manne Children’s Research Institute, Ann & Robert H. Lurie Children’s Hospital of Chicago, Chicago, Illinois; 6 Department of Obstetrics, Gynecology and Reproductive Sciences, University of Pittsburgh, Pittsburgh, Pennsylvania; 7 Department of Epidemiology, University of Pittsburgh, Pittsburgh, Pennsylvania; 8 Department of Cardiology, Cedars-Sinai Medical Center, Los Angeles, California; 9 Department of Public Health and Policy, University of Liverpool, Liverpool, United Kingdom; 10 Department of Obstetrics and Gynecology, The Ohio State University College of Medicine, Columbus

## Abstract

**Importance:**

Hypertensive disorders of pregnancy are leading causes of morbidity and mortality among pregnant individuals as well as newborns, with increasing incidence during the past decade. Understanding the individual associations of advancing age of pregnant individuals at delivery, more recent delivery year (period), and more recent birth year of pregnant individuals (cohort) with adverse trends in hypertensive disorders of pregnancy could help guide public health efforts to improve the health of pregnant individuals.

**Objective:**

To clarify the independent associations of delivery year and birth year of pregnant individuals, independent of age of pregnant individuals, with incident rates of hypertensive disorders of pregnancy.

**Design, Setting, and Participants:**

This serial cross-sectional study of 38 141 561 nulliparous individuals aged 15 to 44 years with a singleton, live birth used 1995-2019 natality data from the National Vital Statistics System.

**Exposures:**

Year of delivery (period) and birth year (cohort) of pregnant individuals.

**Main Outcomes and Measures:**

Rates of incident hypertensive disorders of pregnancy, defined as gestational hypertension, preeclampsia, or eclampsia, recorded on birth certificates. Generalized linear mixed models were used to calculate adjusted rate ratios (aRRs) comparing the incidence of hypertensive disorders of pregnancy in each delivery period (adjusted for age and cohort) and birth cohort (adjusted for age and period) with the baseline group as the reference for each. Analyses were additionally stratified by the self-reported racial and ethnic group of pregnant individuals.

**Results:**

Of 38 141 561 individuals, 20.2% were Hispanic, 0.8% were non-Hispanic American Indian or Alaska Native, 6.5% were non-Hispanic Asian or Pacific Islander, 13.9% were non-Hispanic Black, and 57.8% were non-Hispanic White. Among pregnant individuals who delivered in 2015 to 2019 compared with 1995 to 1999, the aRR for the incidence of hypertensive disorders of pregnancy was 1.59 (95% CI, 1.57-1.62), adjusted for age and cohort. Among pregnant individuals born in 1996 to 2004 compared with 1951 to 1959, the aRR for the incidence of hypertensive disorders of pregnancy was 2.61 (95% CI, 2.41-2.84), adjusted for age and period. The incidence was higher among self-identified non-Hispanic Black individuals in each birth cohort, with similar relative changes for period (aRR, 1.76 [95% CI, 1.70-1.81]) and cohort (aRR, 3.26 [95% CI, 2.72-3.91]) compared with non-Hispanic White individuals (period: aRR, 1.60 [95% CI, 1.57-1.63]; cohort: aRR, 2.53 [95% CI, 2.26-2.83]).

**Conclusions and Relevance:**

This cross-sectional study suggests that more recent birth cohorts of pregnant individuals have experienced a doubling of rates of hypertensive disorders of pregnancy, even after adjustment for age and delivery period. Substantial racial and ethnic disparities persisted across generations.

## Key Points

**Question:**

What is the association of the birth year of pregnant individuals with trends in hypertensive disorders of pregnancy, adjusting for age of pregnant individuals and delivery year between 1995 and 2019?

**Findings:**

In this cross-sectional study of 38 141 561 nulliparous pregnant individuals, the incidence of hypertensive disorders of pregnancy was higher among individuals born in 1996 to 2004 than among individuals born in 1951 to 1959. Rate ratios were similar in each self-reported racial and ethnic group.

**Meaning:**

This study suggests that public health efforts increasing awareness of and focusing on prevention among younger individuals are needed to reverse adverse trends in hypertensive disorders of pregnancy.

## Teaser

This cross-sectional study uses an age-period-cohort analysis to clarify the independent associations of delivery year and birth year of pregnant individuals, independent of age of pregnant individuals, with incident rates of hypertensive disorders of pregnancy.

## Introduction

New-onset hypertensive disorders of pregnancy (HDP), including gestational hypertension, preeclampsia, and eclampsia, are leading causes of morbidity and mortality among pregnant individuals as well as newborns and an important factor associated with the growing health crisis of pregnant individuals in the US.^1^ Emerging evidence also highlights HDP as important “risk-enhancing factors” for lifetime risk of cardiovascular disease in the birthing individual, including a 2- to 4-fold higher risk of heart failure.^2,3^ Rates of HDP have almost doubled during the past decade to approximately 8% of pregnant individuals.^4^ The incidence rate has increased similarly across racial and ethnic groups, and disparities have persisted, with the highest incidence observed among non-Hispanic American Indian or Alaska Native individuals and non-Hispanic Black individuals.^4,5,6^

Age of pregnant individuals at first birth, a known risk factor for HDP, has increased during the same time period and is implicated as a key factor associated with increasing rates of morbidity and mortality among pregnant individuals in the US.^4,7^ However, the incidence of HDP has increased consistently in recent years among all age groups of pregnant individuals, with the greatest relative increases observed among younger pregnant individuals aged 20 to 24 years.^4^ This finding suggests that additional factors associated with all age groups similarly at the time of diagnosis (period factors) and with generations as they move across time (cohort factors) independent of the biological effects of aging (age factors) are associated with adverse trends in HDP.

We therefore used an age-period-cohort analysis to clarify the independent associations of age of pregnant individuals at delivery, year of delivery (period), and birth year of pregnant individuals (cohort) with new-onset HDP among nulliparous individuals. Given known racial and ethnic differences in the incidence of new-onset HDP, we additionally conducted subgroup analyses by self-identified racial and ethnic group of pregnant individuals. Knowledge of the independent associations of age, period, and cohort with trends in HDP overall and in each racial and ethnic group may help guide public health efforts to equitably improve the health of pregnant individuals and their offspring.

## Methods

### Data Source

We used data from the National Vitals Statistics System (NVSS) natality files, which include demographic and health information of birthing individuals and offspring for all live births in the US. Data were recorded onto birth registration records by the professional attendant at birth using information obtained from the birthing individual and medical records.^8^ All data included in this analysis are publicly available online.^9^ We included all individuals aged 15 to 44 years of age with a singleton, first live birth. We restricted our analysis to nulliparous individuals given the known association between nulliparity and higher risk of HDP.^1,10^ We additionally excluded individuals with chronic or prepregnancy hypertension to focus on incidence of new-onset HDP. For this analysis, we defined new-onset HDP based on the NVSS definition as gestational hypertension or preeclampsia with or without a diagnosis of eclampsia. Given that data are deidentified and publicly available, the Northwestern University institutional review board deemed the study exempt from review. Our methods follow the Strengthening the Reporting of Observational Studies in Epidemiology (STROBE) reporting guideline.^11^

### Statistical Analysis

Age-specific new-onset HDP incidence (per 1000 live births) among pregnant individuals was estimated by birth year of pregnant individuals (cohort: 1955-2004) and year of delivery (period: 1995-2019). We created six 5-year age groups (15-19, 20-24, 25-29, 30-34, 35-39, and 40-44 years) and five 5-year delivery periods (1995-1999, 2000-2004, 2005-2009, 2010-2014, and 2015-2019), spanning ten 9-year, partially overlapping cohorts of pregnant individuals referenced by their midpoint (1951-1959 [midpoint, 1955], 1956-1964 [midpoint, 1960], 1961-1969 [midpoint, 1965], 1966-1974 [midpoint, 1970], 1971-1979 [midpoint, 1975], 1976-1984 [midpoint, 1980], 1981-1989 [midpoint, 1985], 1986-1994 [midpoint, 1990], 1991-1999 [midpoint, 1995], and 1996-2004 [midpoint, 2000]). We first used unadjusted rate ratios (RRs) to compare the incidence of new-onset HDP in each 5-year age group with the incidence in the youngest age group (15-19 years); this reference age group was selected because they experienced the lowest incidence of HDP among this sample of nulliparous individuals.

We then used parametric age-period-cohort models to calculate rates in addition to parameters and functions that characterize the roles of age, period, and cohort in the descriptive trends. These models are described in the eMethods in the Supplement and have been extensively described elsewhere.^12,13,14^ We used the age-period-cohort model to estimate the factors associated with period (calendar year at delivery) as RRs comparing incidence of new-onset HDP with the baseline period (1995-1999). We then estimated the net drift—a parameter that approximates the estimated annual percentage change for an age-standardized rate—across 5-year delivery periods (calendar year at delivery) for new-onset HDP in each age group.

Cohort RRs were calculated as incidence ratios adjusted for age and period that compared the incidence of HDP for a given cohort with that in the reference cohort (ie, the 1951-1959 cohort, which experienced the lowest HDP incidence). Cohort RRs are particularly difficult to interpret because cohorts born far apart in time can never be directly compared at the same age. For example, the 1951-1959 cohort was observed for individuals aged 36 to 44 years, while the 1996-2004 cohort was observed for individuals aged 15 to 23 years. Estimation of the RR for any pair of cohorts requires an assumption that is integral to our age-period-cohort model: that the age-specific rates for any pair of cohorts are proportional after adjusting for period (eMethods in the Supplement). Under this assumption, we can estimate the RR for any pair of cohorts as the product of the sequential RRs between the 2 cohorts.^14^ For example, the modeled RR for 1971-1979:1951-1959 would be equal to the product of the RRs for 1971-1979:1966-1974, 1966-1974:1961-1969, 1961-1969:1956-1964, and 1956-1964:1951-1959.

All analyses were conducted overall and stratified by self-reported race and ethnicity group of pregnant individuals (Hispanic, non-Hispanic American Indian or Alaska Native, non-Hispanic Asian or Pacific Islander, non-Hispanic Black, and non-Hispanic White). Owing to large sample sizes, differences in incidence rates and trends were interpreted based on effect sizes and nonoverlapping 95% CIs. Analyses were completed with Stata, version 14.2 (StataCorp LLC) and software from the National Cancer Institute (Surveillance, Epidemiology, and End Results Online Age Period Cohort Analysis Tool).^15^

## Results

### Study Sample

From 1995 to 2019, there were 38 771 194 nulliparous individuals aged 15 to 44 years with a singleton live birth. We excluded 217 015 individuals (0.6%) without available data on HDP and 412 618 individuals (1.1%) with prepregnancy hypertension (eFigure 1 in the Supplement). Of the 38 141 561 individuals included, 20.2% self-identified as Hispanic, 0.8% as non-Hispanic American Indian or Alaska Native, 6.5% as non-Hispanic Asian or Pacific Islander, 13.9% as non-Hispanic Black, and 57.8% as non-Hispanic White. Compared with pregnant individuals from 1995 to 1999, a greater percentage of individuals with a live birth from 2015 to 2019 were aged 30 years or older, identified as Hispanic or non-Hispanic Asian or Pacific Islander, and reported more than a high school education (Table 1).

**Table 1.** Descriptive Statistics for Nulliparous Individuals Aged 15-44 Years With a Singleton, Live Birth in the United States in the First and Last Delivery Period

Characteristic	Delivery period, No. (%)	
	1995-1999	2015-2019
Total	7 675 250 (100)	7 184 354 (100)
Age, y		
15-19	1 857 416 (24.2)	817 199 (11.4)
20-24	2 177 107 (28.4)	1 979 309 (27.6)
25-29	1 935 074 (25.2)	2 054 227 (28.6)
30-34	1 202 128 (15.7)	1 617 078 (22.5)
35-39	428 562 (5.6)	601 317 (8.4)
40-44	74 963 (1.0)	115 215 (1.6)
Race and ethnicity		
Hispanic	1 315 272 (17.1)	1 520 165 (21.2)
Non-Hispanic		
American Indian or Alaska Native	60 253 (0.8)	53 691 (0.8)
Asian or Pacific Islander	362 173 (4.7)	585 288 (8.2)
Black	1 077 032 (14.0)	1 013 541 (14.1)
White	4 765 935 (62.1)	3 941 381 (54.9)
No response	94 585 (1.2)	70 279 (1.0)
Educational level		
High school or less	4 033 246 (52.6)	2 436 598 (33.9)
Greater than high school	3 539 246 (46.1)	4 634 594 (64.5)
No response	102 758 (1.3)	113 152 (1.6)
Initiation of prenatal care		
During first trimester	6 309 024 (82.2)	5 516 115 (76.8)
During second trimester	952 859 (12.4)	1 057 779 (14.7)
During third trimester	178 672 (2.3)	305 666 (4.3)
None	62 352 (0.8)	97 083 (1.4)
No response	172 343 (2.3)	207 702 (2.9)

### Association of Age of Pregnant Individuals With Trends in New-Onset HDP Rates

**Figure 1.**
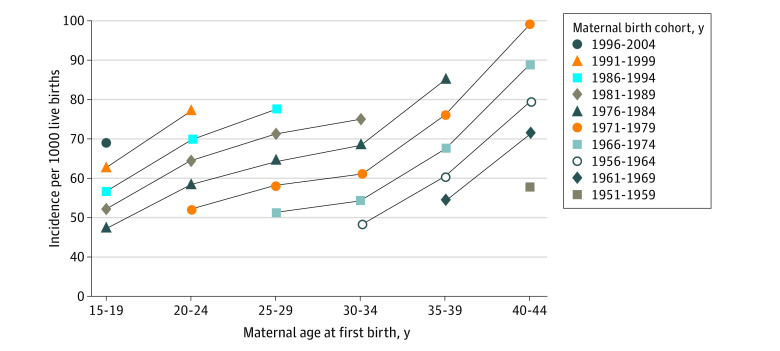
Estimated Incidence of New-Onset Hypertensive Disorders of Pregnancy by Age of Pregnant Individuals at Delivery in Each Cohort, Adjusted for Nonlinear Period Factors Incidence of new-onset hypertensive disorders of pregnancy per 1000 live births is higher with older age of pregnant individuals and more recent birth cohort of pregnant individuals, after adjustment for nonlinear period factors.

Incidence of new-onset HDP was higher among groups with older age of pregnant individuals (Figure 1; Table 2; eTable 1 in the Supplement). Specifically, individuals aged 40 to 44 years at the time of delivery were 1.49 (95% CI, 1.47-1.50) times more likely to develop new-onset HDP than individuals aged 15 to 19 years (eTable 1 in the Supplement) when pooled across the entire study period (calendar years of delivery, 1995-2019). Within each delivery period and cohort (birth year of pregnant individuals), the incidence of HDP was also higher with older age of pregnant individuals. Similar patterns were observed among all racial and ethnic groups (eTables 1-3 and eFigure 2 in the Supplement). In the most recent period (calendar years of delivery, 2015-2019) and cohort (birth year of pregnant individuals, 1996-2004), the incidence in each age group was higher among individuals who identified as non-Hispanic American Indian or Alaska Native and non-Hispanic Black (eTable 2 and eTable 3 in the Supplement). Rate ratios comparing pregnant individuals aged 40 to 44 years with those aged 15 to 19 years were higher among individuals who identified as Hispanic or Latina (RR, 1.81 [95% CI, 1.76-1.86]), non-Hispanic American Indian or Alaska Native (RR, 2.21 [95% CI, 1.95-2.49]), non-Hispanic Asian or Pacific Islander (RR, 1.96 [95% CI, 1.87-2.05]), and non-Hispanic Black, (RR, 1.72 [95% CI, 1.67-1.77]) than among individuals who identified as non-Hispanic White (RR, 1.38 [95% CI, 1.37-1.40]) (eTable 1 in the Supplement).

**Table 2.** Number of Pregnant Individuals in Each Age Group, Period, and Cohort and Unadjusted Incidence of New-Onset HDP per 1000 Live Births

Period (year of delivery)	Pregnant individuals, No. (%) [unadjusted incidence of new-onset HDP per 1000 live births] (N = 38 141 561)					
	Aged 15-19 y at delivery	Aged 20-24 y at delivery	Aged 25-29 y at delivery	Aged 30-34 y at delivery	Aged 35-39 y at delivery	Aged 40-44 y at delivery
1995-1999	1 852 637 (4.7) [52.2]^a^	2 166 885 (5.5) [57.0]^b^	1 921 602 (4.9) [55.7]^c^	1 190 362 (3.0) [53.4]^d^	421 552 (1.1) [60.2]^e^	73 051 (1.9) [68.4]^f^
2000-2004	1 682 597 (4.3) [50.9]^g^	2 318 174 (5.9) [57.1]^a^	1 873 442 (4.8) [57.1]^b^	1 314 305 (3.4) [54.0]^c^	486 409 (1.2) [59.2]^d^	94 153 (0.2) [67.5]^e^
2005-2009	1 691 178 (4.3) [50.1]^h^	2 488 658 (6.3) [57.3]^g^	2 045 852 (5.2) [57.8]^a^	1 284 533 (3.3) [53.7]^b^	514 853 (1.3) [59.8]^c^	102 755 (0.3) [68.1]^d^
2010-2014	1 230 500 (3.1) [56.3]^i^	2 245 970 (5.7) [62.9]^h^	2 044 785 (5.2) [64.2]^g^	1 425 583 (3.6) [61.1]^a^	499 935 (1.3) [68.3]^b^	107 368 (0.3) [81.9]^c^
2015-2019	811 476 (2.1) [79.0]^j^	1 955 987 (5.0) [88.5]^i^	2 021 596 (5.2) [88.5]^h^	1 584 080 (4.0) [85.7]^g^	581 676 (1.5) [97.8]^a^	109 607 (0.3) [116.9]^b^

Abbreviation: HDP, hypertensive disorders of pregnancy.

^a^ Corresponds to 1976-1984 birth cohort.

^b^ Corresponds to 1971-1979 birth cohort.

^c^ Corresponds to 1966-1974 birth cohort.

^d^ Corresponds to 1961-1969 birth cohort.

^e^ Corresponds to 1956-1964 birth cohort.

^f^ Corresponds to 1951-1959 birth cohort.

^g^ Corresponds to 1981-1989 birth cohort.

^h^ Corresponds to 1986-1994 birth cohort.

^i^ Corresponds to 1990-1999 birth cohort.

^j^ Corresponds to 1996-2004 birth cohort.

### Association of Period With Trends in New-Onset HDP Rates

Incidence of new-onset HDP increased across the entire study period (calendar year of delivery, 1995-2019), with an annual mean percentage change of 2.1% per year (95% CI, 2.1%-2.2% per year) after adjustment for age of pregnant individuals at delivery and cohort (birth year of pregnant individuals). Period RRs were initially stable (1995-2009) and increased during the past decade (2010-2019). Pregnant individuals who delivered during the 2010-2014 and 2015-2019 periods were 1.13 (95% CI, 1.11-1.14) and 1.59 (95% CI, 1.57-1.62) times more likely, respectively, to experience a new-onset HDP than those who delivered during the baseline period (1995-1999) (Table 3). In each racial and ethnic group, the incidence also increased from 1995-1999 to 2015-2019 (eTable 1 in the Supplement). Across racial and ethnic groups, the period RRs comparing the incidence of new-onset HDP in pregnant individuals who delivered in the 2015-2019 period with those who delivered in the referent period (1995-1999) were significantly higher among pregnant individuals who identified as Hispanic (1.73 [95% CI, 1.65-1.82]), non-Hispanic Black (1.76 [95% CI, 1.70-1.81]), and non-Hispanic Asian or Pacific Islander (1.81 [95% CI, 1.73-1.89]) than among those who identified as non-Hispanic American Indian or Alaska Native (1.48 [95% CI, 1.38-1.59]) or non-Hispanic White (1.60 [95% CI, 1.57-1.63]) (Table 3).

**Table 3.** Rate Ratios of New-Onset Hypertensive Disorders of Pregnancy Overall and in Each Racial and Ethnic Group^a^

Year	Rate ratio (95% CI)					
	Overall	Hispanic	Non-Hispanic			
			American Indian or Alaska Native	Asian or Pacific Islander	Black	White
**Period (calendar year of delivery)^b^**						
1995-1999	1 [Reference]	1 [Reference]	1 [Reference]	1 [Reference]	1 [Reference]	1 [Reference]
2000-2004	1.00 (0.99-1.01)	0.95 (0.92-0.98)	0.95 (0.91-0.99)	0.98 (0.94-1.02)	1.02 (1.00-1.04)	1.03 (1.01-1.04)
2005-2009	1.00 (0.99-1.01)	0.98 (0.94-1.01)	0.98 (0.94-1.03)	1.02 (0.98-1.07)	1.09 (1.06-1.11)	1.02 (1.00-1.04)
2010-2014	1.13 (1.11-1.14)	1.17 (1.13-1.22)	1.03 (0.98-1.10)	1.20 (1.15-1.25)	1.25 (1.22-1.29)	1.13 (1.11-1.15)
2015-2019	1.59 (1.57-1.62)	1.73 (1.65-1.82)	1.48 (1.38-1.59)	1.81 (1.73-1.89)	1.76 (1.70-1.81)	1.60 (1.57-1.63)
**Cohort (birth year of pregnant individuals)^c^**						
1951-1959	1 [Reference]	1 [Reference]	1 [Reference]	1 [Reference]	1 [Reference]	1 [Reference]
1956-1964	1.14 (1.05-1.25)	1.31 (0.97-1.77)	0.93 (0.62-1.39)	1.06 (0.87-1.29)	1.15 (0.95-1.39)	1.12 (1.01-1.26)
1961-1969	1.27 (1.17-1.38)	1.46 (1.09-1.96)	1.09 (0.74-1.59)	1.15 (0.95-1.39)	1.30 (1.08-1.56)	1.25 (1.12-1.39)
1966-1974	1.42 (1.31-1.54)	1.66 (1.24-2.21)	1.19 (0.82-1.75)	1.29 (1.07-1.56)	1.47 (1.23-1.76)	1.41 (1.27-1.57)
1971-1979	1.60 (1.47-1.73)	1.89 (1.42-2.53)	1.35 (0.93-1.97)	1.49 (1.24-1.79)	1.69 (1.41-2.02)	1.62 (1.46-1.80)
1976-1984	1.79 (1.65-1.94)	2.19 (1.64-2.92)	1.47 (1.01-2.15)	1.77 (1.47-2.13)	1.96 (1.64-2.35)	1.80 (1.62-2.01)
1981-1989	1.97 (1.82-2.14)	2.49 (1.86-3.33)	1.60 (1.09-2.34)	2.05 (1.70-2.47)	2.24 (1.87-2.68)	1.97 (1.77-2.19)
1986-1994	2.14 (1.97-2.32)	2.78 (2.08-3.72)	1.73 (1.18-2.52)	2.28 (1.89-2.75)	2.52 (2.11-3.02)	2.10 (1.89-2.34)
1991-1999	2.37 (2.19-2.57)	3.15 (2.36-4.21)	1.77 (1.21-2.60)	2.79 (2.30-3.39)	2.88 (2.41-3.45)	2.29 (2.06-2.56)
1996-2004	2.61 (2.41-2.84)	3.41 (2.55-4.57)	1.90 (1.29-2.78)	3.33 (2.64-4.19)	3.26 (2.72-3.91)	2.53 (2.26-2.83)

^a^ Rate ratios compared incidence of new-onset hypertensive disorders of pregnancy with the baseline period (calendar year of delivery, 1995-1999) and baseline cohort (birth year of pregnant individuals, 1951-1959).

^b^ Adjusted for age of pregnant individuals at delivery and nonlinear birth cohort factors.

^c^ Adjusted for age of pregnant individuals at delivery and nonlinear period factors.

### Association of Cohort With Trends in New-Onset HDP

**Figure 2.**
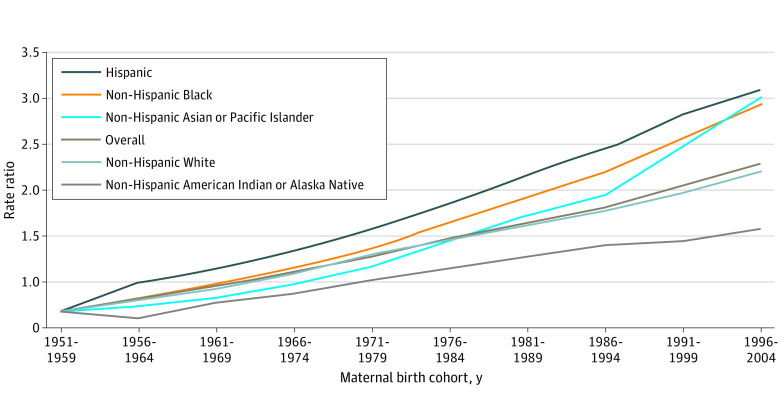
Adjusted Cohort Rate Ratios for Incidence of New-Onset Hypertensive Disorders of Pregnancy Compared With the Baseline Cohort Overall and Among Each Race and Ethnicity Group Rate ratios were adjusted for age and period (calendar year of delivery). Cohort rate ratios are higher among more recent birth cohorts of pregnant individuals in each racial and ethnic group after adjustment for age of pregnant individuals and nonlinear period factors.

Later birth year of pregnant individuals (more recent birth cohort) was associated with a higher incidence of new-onset HDP than earlier birth year of pregnant individuals, even after adjusting for age and period (Figure 1; Table 3). Specifically, pregnant individuals born in the 1996 to 2004 cohort were 2.61 (95% CI, 2.41-2.84) times more likely to experience new-onset HDP compared with pregnant individuals who were born in the 1951 to 1959 cohort (Table 3; Figure 2). Birth cohort RRs were higher among self-identified Hispanic individuals (3.41 [95% CI, 2.55-4.57]), non-Hispanic Black individuals (3.26 [95% CI, 2.72-3.91]), and non-Hispanic Asian or Pacific Islander individuals (3.33 [95% CI, 2.64-4.19]) compared with non-Hispanic American Indian or Alaska Native individuals (1.90 [1.29-2.78]) and non-Hispanic White individuals (2.53 [95% CI, 2.26-2.83]), although 95% CIs overlapped (Table 3; Figure 2).

## Discussion

This nationwide age-period-cohort analysis demonstrated 3 key findings: The incidence of new-onset HDP was higher among pregnant individuals with (1) older age at delivery, (2) more recent period (calendar year of delivery), and (3) more recent cohort (birth year of pregnant individuals). Nulliparous pregnant individuals who were born in more recent years experienced significantly higher rates of new-onset HDP, even after adjustment for age of pregnant individuals and calendar year at delivery, which accounts for secular period factors. Specifically, pregnant individuals born in the 1990s and 2000s were more than twice as likely than those born in the 1950s to have new-onset HDP. Persistent racial and ethnic differences in the incidence of new-onset HDP were observed across age of pregnant individuals, delivery period, and birth cohort, with the highest rates observed among self-identified non-Hispanic American Indian or Alaska Native individuals and non-Hispanic Black individuals.

The present study expands on prior analyses that previously demonstrated increases in the incidence of HDP across age groups of pregnant individuals and delivery periods by additionally considering the interrelated factors of age, period, and cohort simultaneously.^4,16,17,18^ We demonstrate that the incidence of HDP has increased during the past 2 decades, with accelerating rates from 2010 to 2019, similar to prior studies,^4,17^ but now additionally controlling for factors associated with birth cohort. In addition, we add to the literature by describing the independent association of birth cohort of pregnant individuals with trends in rates of HDP among nulliparous individuals in the US between 1995 and 2019. Our findings thus complement and build on previously published data, such as an analysis from the National Inpatient Sample from 1980 to 2010. That analysis included only a subset of delivery hospitalizations in the US and similarly demonstrated that pregnant individuals born in the 1970 birth cohort were 1.2 times more likely than those born in the 1955 birth cohort to have preeclampsia.^18^ Our analysis, which used a contemporary sample of live births in the US up to 2019, included all new-onset cases of HDP and all live births (both within and outside the hospital setting).

Although our study is ecological and cannot identify factors associated with trends, increasing incidence of HDP among younger generations may reflect adverse trends in preconception health. Poor cardiovascular health prior to pregnancy is an important risk factor for HDP, as well as other important outcomes, including severe morbidity of pregnant individuals, mortality of pregnant individuals, and cardiovascular health of offspring into adolescence.^19,20,21,22,23,24^ In the Hyperglycemia and Adverse Pregnancy Outcome Study, pregnant individuals with poor cardiovascular health at a mean gestational age of 28 weeks were more than 9 times more likely than those with ideal cardiovascular health to develop preeclampsia.^24^ A recent report from the NVSS highlighted the decline in preconception health among pregnant individuals, with the overall percentage of individuals in the US entering pregnancy with favorable cardiovascular health decreasing from 42.1% to 37.7% between 2011 and 2019.^20^ Among the components of cardiovascular health, obesity has had the largest increase in prevalence from 6.2% in the 1976-1980 period to 32.7% in the 2017-2018 period.^25^ The population-attributable fraction for HDP associated with obesity is estimated to be between 25% and 30% and has not changed significantly between 2011 and 2019,^21^ which suggests that obesity may be only partially associated with trends in HDP. Given that data on obesity and other cardiovascular health factors and behaviors were not available in the present study, further detailed work with pregnancy-specific cohorts with long-term follow-up is needed to understand the association of the obesity epidemic, as well as the decline in prepregnancy cardiovascular health, with trends in HDP and to inform strategies to target morbidity and mortality among pregnant individuals, as highlighted by the recent call to action policy statement by the American Heart Association and supported by the American College of Obstetrics and Gynecology.^26^

The increasing rates of HDP among younger individuals from more recent generations, as shown in our study, highlight the urgency with which these strategies need to be implemented. At the clinical level, increased awareness of adverse trends in HDP among younger generations informs practitioners about the importance of HDP prevention and management among individuals of all ages, not only among older individuals. Assessing cardiovascular risk early in the life course and developing strategies with patients to optimize cardiovascular health prior to a first pregnancy could have an effect on reducing the incidence of HDP. Multidisciplinary teams composed of primary care physicians, cardiologists, and obstetricians-gynecologists are needed to comprehensively manage HDP risk factors before, during, and after pregnancy.

We also identified significant associations with birth cohort among all racial and ethnic groups and demonstrated that racial and ethnic disparities have persisted across 4 generations. The present analyses specifically inform a growing body of work focusing on the root structural causes of racial and ethnic differences in adverse health outcomes of pregnant individuals and infants.^27,28,29^ The present study is an epidemiologic analysis of trends and is, therefore, not well suited to examine causal factors, such as social or structural determinants of health, that may be associated with trends and disparities. Prior work has implicated residential segregation and racism in race- and ethnicity-based disparities, with greater rates of HDP among pregnant individuals residing in more racially and ethnically segregated areas.^30,31^ Race- and ethnicity-based differences in related birth outcomes (preterm birth and low birth weight) have also been associated with individual-level reports of racial and ethnic discrimination as well as neighborhood-level factors, such as census-block reports of excessive use of force by law enforcement officers.^32,33^

Our analyses also demonstrate greater RRs for the incidence of new-onset HDP among older pregnant individuals at delivery who did not identify as White. This dynamic was associated with widening racial and ethnic gaps for new-onset HDP among older age groups. This may be partially explained by the weathering hypothesis, which postulates that groups with cumulative exposure to systemic and structural disadvantage experience an acceleration in the deterioration of health.^34^ Previous data from the National Center for Health Statistics demonstrated that from 2014 to 2017, the gap between Black and White individuals in the delivery of low-birth-weight infants was 2-fold higher across age, from 5.2% among 15-year-old individuals to 10.0% among 40-year-old individuals.^28^ Because these disparities likely reflect upstream social determinants, including structural barriers perpetuated by systemic racism, focusing policy efforts on social factors (eg, income, food insecurity, nutritional insecurity, green space availability, health literacy, and access to health care)^26,35^ could have a meaningful effect on equitably improving health among pregnant individuals from all age groups.

### Limitations

The limitations of this work should be noted. There is the potential for misclassification of a diagnosis of new-onset HDP based on birth registration records in the NVSS. However, the NVSS birth data represent the most robust source of national surveillance of birth records and include data from more than 38 million individuals with rates similar to those identified in administrative hospitalization data sets.^36^ In addition, birth certificates were revised in 2003 to include additional risk factors of pregnant individuals and pregnancy complications, as well as a standardized worksheet to assist pregnant individuals and hospital staff with birth certificate completion. Although it is possible that these changes may have influenced the reporting and classification of HDP, the Centers for Disease Control and Prevention User Guide to the natality Public Use File reported these variables as comparable across these birth certificate revisions.^37^ In 2013, the definition of preeclampsia was revised by the American College of Obstetricians and Gynecologists to include pregnancies that otherwise met criteria for preeclampsia even if proteinuria was not present.^10^ By using a composite definition of HDP that included both gestational hypertension and preeclampsia, we mitigated the potential association of this changing definition with trends in HDP. Furthermore, birth cohort trends of pregnant individuals were adjusted for the factors associated with delivery period that accounted for changing practice patterns at the time of delivery. Given that available NVSS data combine, and do not distinguish between, gestational hypertension and preeclampsia, we were unable to investigate these trends separately. However, because gestational hypertension and preeclampsia are managed similarly, the combined definition of new-onset HDP represents a clinically relevant outcome for the study. In addition, this study is ecological in nature, and, therefore, the goal is not to identify causes of the observed trends and disparities; also, data on potential risk factors for HDP, such as diet, physical activity, and body mass index, were not available from all states across the duration of the study period.

## Conclusions

This study found that the incidence of new-onset HDP among nulliparous pregnant individuals has more than doubled for those born in the 1990s and 2000s compared with those born in the 1950s, even after adjustment for age of pregnant individuals and delivery period. Furthermore, racial and ethnic disparities in new-onset HDP have persisted without narrowing across 4 generations. This work underscores the need for future investigations to identify potential interventions to reduce the frequency of HDP and guide public health reforms to equitably improve the health of pregnant individuals.

## Notes

Supplement.
eMethods.

eReferences.
**eTable 1.** Rate Ratios Comparing Unadjusted Incidence of New-Onset Hypertensive Disorders of Pregnancy to the Reference Age Group (15-19 Year Olds) **eTable 2.** Estimated Incidence of New-Onset Hypertensive Disorders of Pregnancy (per 1000 Live Births) in Each Maternal Age at Delivery, Race and Ethnicity Group in the First (1995-1999) and Last (2015-2019) Period (Calendar Year of Delivery) Adjusted for Non-Linear Cohort (Maternal Birth Year) Effects **eTable 3.** Estimated Incidence of New-Onset Hypertensive Disorders of Pregnancy (per 1000 Live Births) in Each Maternal Age, Race and Ethnicity Group in the First (1951-1959) and Last (1996-2004) Cohort (Maternal Birth Year) Adjusted for Non-Linear Period (Calendar Year of Delivery) Effects **eFigure 1.** Study Population Utilized From the National Vital Statistics System **eFigure 2.** Estimated Incidence of New-Onset Hypertensive Disorders of Pregnancy (per 1000 Live Births) in Each Maternal Age at Delivery, Race and Ethnicity in the First (1951-1959) and Last (1996-2004) Cohort (Maternal Birth Year) Adjusted for Non-Linear Period (Calendar Year of Delivery) Effects
