# Supplementary material for: Association of Birth Year of Pregnant Individuals With Trends in Hypertensive Disorders of Pregnancy in the United States, 1995-2019
Source: JAMA Netw Open. 2022 Aug 24;5(8):e2228093. doi: 10.1001/jamanetworkopen.2022.28093 (PMC9403773; doi:10.1001/jamanetworkopen.2022.28093)
Supplement: Supplement. — eMethods. eReferences. eTable 1. Rate Ratios Comparing Unadjusted Incidence of New-Onset Hypertensive Disorders of Pregnancy to the Reference Age Group (15-19 Year Olds) eTable 2. Estimated Incidence of New-Onset Hypertensive Disorders of Pregnancy (per 1000 Live Births) in Each Maternal Age at Delivery, Race and Ethnicity Group in the First (1995-1999) and Last (2015-2019) Period (Calendar Year of Delivery) Adjusted for Non-Linear Cohort (Maternal Birth Year) Effects eTable 3. Estimated Incidence of New-Onset Hypertensive Disorders of Pregnancy (per 1000 Live Births) in Each Maternal Age, Race and Ethnicity Group in the First (1951-1959) and Last (1996-2004) Cohort (Maternal Birth Year) Adjusted for Non-Linear Period (Calendar Year of Delivery) Effects eFigure 1. Study Population Utilized From the National Vital Statistics System eFigure 2. Estimated Incidence of New-Onset Hypertensive Disorders of Pregnancy (per 1000 Live Births) in Each Maternal Age at Delivery, Race and Ethnicity in the First (1951-1959) and Last (1996-2004) Cohort (Maternal Birth Year) Adjusted for Non-Linear Period (Calendar Year of Delivery) Effects [file jamanetwopen-e2228093-s001.pdf]

Supplementary Online Content

Cameron NA, Petito LC, Shah NS, et al. Association of birth year of pregnant individuals with trends in hypertensive disorders of pregnancy in the United States, 1995-2019. *JAMA Netw Open.* 2022;5(8):e2228093. doi:10.1001/jamanetworkopen.2022.28093

eMethods.
eReferences.
eTable 1. Rate Ratios Comparing Unadjusted Incidence of New-Onset Hypertensive Disorders of Pregnancy to the Reference Age Group (15-19 Year Olds)
eTable 2. Estimated Incidence of New-Onset Hypertensive Disorders of Pregnancy (per 1000 Live Births) in Each Maternal Age at Delivery, Race and Ethnicity Group in the First (1995-1999) and Last (2015-2019) Period (Calendar Year of Delivery) Adjusted for Non-Linear Cohort (Maternal Birth Year) Effects
eTable 3. Estimated Incidence of New-Onset Hypertensive Disorders of Pregnancy (per 1000 Live Births) in Each Maternal Age, Race and Ethnicity Group in the First (1951-1959) and Last (1996-2004) Cohort (Maternal Birth Year) Adjusted for Non-Linear Period (Calendar Year of Delivery) Effects
eFigure 1. Study Population Utilized From the National Vital Statistics System
eFigure 2. Estimated Incidence of New-Onset Hypertensive Disorders of Pregnancy (per 1000 Live Births) in Each Maternal Age at Delivery, Race and Ethnicity in the First (1951-1959) and Last (1996-2004) Cohort (Maternal Birth Year) Adjusted for Non-Linear Period (Calendar Year of Delivery) Effects

This supplementary material has been provided by the authors to give readers additional information about their work.

© 2022 Cameron NA et al. *JAMA Network Open.*

eMethods.

Identifiability in APC Modeling

Identifiability in APC analyses, or whether log-linear trends in rates can be attributed to age, period, or cohort, is a well-known and documented issue.[1] Holford first demonstrated mathematically that additional unverifiable assumptions are needed for identifiability, as cohort equals period minus age.[2–4] Because the age, period, and cohort variables are collinear, it is not possible to completely separate the linear trend in calendar period effects from the linear trend in age effects, or the linear trend in birth cohort effects from the linear trend in calendar period effects. However, Rosenberg and Anderson argue that these issues are implicit in all cohort analyses, and often skated over due to eligibility constraints placed on the cohorts at hand (e.g. restricting cohort eligibility to a narrow age window).[5–7] Because the data we have used, US birth certificates, cover a fairly large time window (25 years) and the period of life we are concerned with is sufficiently short (childbearing years), we believe these data contain enough breadth and depth to reveal long-term secular trends in the incidence of HDP in the population associated with age, period, and cohort. Furthermore, certain age-period-cohort parameters can be estimated if the age, calendar period, and birth cohort trends are orthogonally decomposed into their linear and nonlinear components.

Model Specification

We view APC modeling as a useful parametric complement to standard nonparametric descriptive methods.[1,3,8,9] This age-period-cohort (APC) analysis strives to quantify the independent effects of age, period, and cohort on trends in hypertensive disorder of pregnancy (HDP) while addressing the inherent collinearity among them. To be precise about our formulation of the underlying statistical model, we first introduce some notation.[6] We define two matrices: $\boldsymbol{Y} = [Y_{pa}\ where\ p = 1, \ldots, P;\ a = 1, \ldots, A]$ that contains the number of diagnoses of HDP in calendar period p and age group a, and $\boldsymbol{O} = [O_{pa}\ where\ p = 1, \ldots, P;\ a = 1, \ldots, A]$ that contains the corresponding number of live births. The observed incidence rates per 1,000 person-years are $\lambda_{pa} = 10^3 Y_{pa}/O_{pa}$, and the corresponding expected log rates are $\rho_{pa} = \log\,[E(Y_{pa})/O_{pa}]$.

APC analysis is based on a log-linear model for the expected rates with additive effects for age, period, and cohort:

$$\rho_{pa} = \alpha_a + \pi_p + \gamma_c,$$

where ρ_{pa} is the expected log rate for calendar period p and age group a, and α_a, π_p, and γ_c are generic additive effects. This equation can be partitioned into linear and nonlinear components, while incorporating the fundamental constraint that $c = p - a + A$.[3,6] Two frequently used forms are the age-period form:

$$\rho_{pa} = \mu + (\alpha_L - \gamma_L)(a - \bar{a}) + \overline{\alpha_a} + (\pi_L + \gamma_L)(p - \bar{p}) + \overline{\pi_p} + \overline{\gamma_{p-a+A}}, \qquad (1)$$

and the age-cohort form:

$$\rho_{ca} = \mu + (\alpha_L + \pi_L)(a - \bar{a}) + \overline{\alpha_a} + (\pi_L + \gamma_L)(c - \bar{c}) + \overline{\pi_{c-a+A}} + \overline{\gamma_c}. \qquad (2)$$

Our final models took the form of equations (1) and (2). Although the initial model is not identifiable, each of the terms in equations (1) and (2) is identifiable. The parameter μ is the intercept term, and the parameters $\overline{\alpha_a}\,, \overline{\pi_p}, and\ \overline{\gamma_c}$ are the orthogonal deviations as presented originally by Holford[3].

From these equations, we estimated the following parameters and functions. The *net drift* is the APC analog of the estimated annual percent change of the age standardized rate – it represents the average annual percentage change in incidence per calendar year, and in our parameterization is estimated as $\pi_L + \gamma_L$ (note: this quantity appears in both equations 1 and 2).[8,10,11] The *period rate ratios* are the ratio of age-specific rates in period p relative to reference period $\bar{p}$ (from equation 1), adjusted for any cohort effects influencing all age groups simultaneously. The *cohort rate ratio* curves are the ratio of age-specific rates in cohort c relative to reference cohort $\bar{c}$ (from equation 2), adjusted for any calendar period effects influencing all age groups simultaneously.

© 2022 Cameron NA et al. *JAMA Network Open.*

eReferences.

1. Smith TR, Wakefield J. A Review and Comparison of Age–Period–Cohort Models for Cancer Incidence. *SSO Schweiz Monatsschr Zahnheilkd*. 2016;31(4):591-610.

2. Holford TR. Understanding the effects of age, period, and cohort on incidence and mortality rates. *Annu Rev Public Health*. 1991;12:425-457.

3. Holford TR. The estimation of age, period and cohort effects for vital rates. *Biometrics*. 1983;39(2):311-324.

4. Holford TR. Age-period-cohort analysis. In: Armitage P, Colton T, eds. *Encyclopedia of Biostatistics*. Vol 1. 2. John Wiley & Sons; 2005:105-123.

5. Rosenberg PS, Check DP, Anderson WF. A web tool for age-period-cohort analysis of cancer incidence and mortality rates. *Cancer Epidemiol Biomarkers Prev*. 2014;23(11):2296-2302.

6. Rosenberg PS, Anderson WF. Age-period-cohort models in cancer surveillance research: ready for prime time? *Cancer Epidemiol Biomarkers Prev*. 2011;20(7):1263-1268.

7. Robertson C, Boyle P. Age-period-cohort models of chronic disease rates. II: Graphical approaches. *Stat Med*. 1998;17(12):1325-1339.

8. Clayton D, Schifflers E. Models for temporal variation in cancer rates. II: Age-period-cohort models. *Stat Med*. 1987;6(4):469-481.

9. Carstensen B. Age-period-cohort models for the Lexis diagram. *Stat Med*. 2007;26(15):3018-3045.

10. Robertson C, Boyle P. Age-period-cohort analysis of chronic disease rates. I: Modelling approach. *Stat Med*. 1998;17(12):1305-1323.

11. Anderson WF, Rosenberg PS, Menashe I, Mitani A, Pfeiffer RM. Age-related crossover in breast cancer incidence rates between black and white ethnic groups. *J Natl Cancer Inst*. 2008;100(24):1804-1814.

© 2022 Cameron NA et al. *JAMA Network Open.*

eTable 1. Rate Ratios Comparing Unadjusted Incidence of New-Onset Hypertensive Disorders of Pregnancy to the Reference Age Group (15-19 Year Olds)

			Non-Hispanic			
	**Overall**	**Hispanic**	**American Indian/ Alaskan Native**	**Asian/Pacific Islander**	**Black**	**White**
**N**	**38,141,561**	**7,693,778**	**312,301**	**2,472,105**	**5,283,903**	**22,037,764**
**Maternal Age (years)**	**RATE RATIOS (95% Confidence Intervals)**					
15-19	1 (reference)	1 (reference)	1 (reference)	1 (reference)	1 (reference)	1 (reference)
20-24	1.16 (1.15-1.16)	1.08 (1.08-1.09)	1.17 (1.14-1.21)	0.98 (0.95-1.02)	1.14 (1.14-1.15)	1.19 (1.18-1.19)
25-29	1.18 (1.17-1.18)	1.15 (1.14-1.16)	1.29 (1.25-1.34)	0.88 (0.85-0.91)	1.24 (1.23-1.25)	1.20 (1.19-1.21)
30-34	1.14 (1.13-1.14)	1.25 (1.23-1.26)	1.40 (1.34-1.47)	1.04 (1.00-1.08)	1.31 (1.30-1.33)	1.13 (1.12-1.13)
35-39	1.28 (1.27-1.28)	1.47 (1.45-1.49)	1.64 (1.54-1.75)	1.45 (1.40-1.50)	1.47 (1.45-1.50)	1.22 (1.22-1.23)
40-44	1.49 (1.47-1.50)	1.81 (1.76-1.86)	2.21 (1.95-2.49)	1.96 (1.87-2.05)	1.72 (1.67-1.77)	1.38 (1.37-1.40)

© 2022 Cameron NA et al. *JAMA Network Open.*

eTable 2. Estimated Incidence of New-Onset Hypertensive Disorders of Pregnancy (per 1000 live Births) in each Maternal Age at Delivery, Race and Ethnicity Group in the First (1995-1999) and Last (2015-2019) Period (Calendar year of Delivery) Adjusted for Non-Linear Cohort (Maternal Birth Year) Effects

**Age (years) -Race/Ethnicity Group**	**Period (Calendar Year of Delivery)**	
	**1995-1999**	**2015-2019**
**Age 15-19 years**		
Hispanic	39.74 (38.68, 40.83)	68.88 (65.96, 71.93)
Non-Hispanic American Indian/Alaskan Native	69.83 (67.45, 72.30)	103.32 (97.16, 109.89)
Non-Hispanic Asian/Pacific Islander	28.00 (26.38, 29.72)	50.70 (47.82, 53.75)
Non-Hispanic Black	54.09 (53.11, 55.08)	94.99 (92.33, 97.73)
Non-Hispanic White	54.86 (53.94, 55.80)	87.88 (86.02, 89.79)
**Age 20-24 years**		
Hispanic	40.78 (39.57, 42.04)	70.69 (68.44, 73.01)
Non-Hispanic American Indian/Alaskan Native	78.56 (75.35, 81.91)	116.24 (110.95, 121.78)
Non-Hispanic Asian/Pacific Islander	26.28 (25.21, 27.39)	47.58 (45.73, 49.51)
Non-Hispanic Black	57.72 (56.55, 58.92)	101.38 (99.20, 103.61)
Non-Hispanic White	62.47 (61.56, 63.39)	100.06 (98.46, 101.70)

© 2022 Cameron NA et al. *JAMA Network Open.*

Age (years) -Race/Ethnicity Group	Period (Calendar Year of Delivery)	
**Age 25-29 years**	**1995-1999**	**2015-2019**
Hispanic	42.27 (40.66, 43.96)	73.27 (71.35, 75.24)
Non-Hispanic American Indian/Alaskan Native	83.83 (79.23, 88.70)	124.03 (119.11, 129.16)
Non-Hispanic Asian/Pacific Islander	23.07 (22.22, 23.95)	41.77 (40.51, 43.07)
Non-Hispanic Black	61.05 (59.47, 62.68)	107.23 (105.22, 109.28)
Non-Hispanic White	61.70 (60.70, 62.71)	98.83 (97.50, 100.18)
**Age 30-34 years**		
Hispanic	44.55 (42.42, 46.78)	77.21 (75.06, 79.42)
Non-Hispanic American Indian/Alaskan Native	91.20 (84.87, 98.01)	134.94 (128.6, 141.59)
Non-Hispanic Asian/Pacific Islander	26.04 (25.01, 27.10)	47.15 (45.92, 48.40)
Non-Hispanic Black	64.40 (62.35, 66.53)	113.11 (110.74, 115.54)
Non-Hispanic White	56.98 (55.87, 58.11)	91.28 (90.03, 92.55)
**Age 35-39 years**		
Hispanic	51.88 (48.83, 55.12)	89.92 (86.39, 93.59)
Non-Hispanic American Indian/Alaskan Native	108.12 (98.84, 118.26)	159.96 (148.92, 171.83)

© 2022 Cameron NA et al. *JAMA Network Open.*

Age (years) -Race/Ethnicity Group	Period (Calendar Year of Delivery)	
**Age 35-39 years**	**1995-1999**	**2015-2019**
Non-Hispanic Asian/Pacific Islander	36.11 (34.46, 37.84)	65.38 (63.40, 67.42)
Non-Hispanic Black	72.36 (69.53, 75.31)	127.08 (123.39, 130.9)
Non-Hispanic White	62.45 (60.93, 64.01)	100.04 (98.09, 102.04)
**Age 40-44 years**		
Hispanic	64.12 (58.92, 69.77)	111.13 (102.93, 119.97)
Non-Hispanic American Indian/Alaskan Native	145.81 (127.2, 167.14)	215.73 (187.68, 247.98)
Non-Hispanic Asian/Pacific Islander	48.34 (45.37, 51.51)	87.53 (82.86, 92.47)
Non-Hispanic Black	84.20 (79.51, 89.16)	147.88 (139.92, 156.29)
Non-Hispanic White	72.05 (69.30, 74.91)	115.42 (110.93, 120.09)

© 2022 Cameron NA et al. *JAMA Network Open.*

eTable 3. Estimated Incidence of New-Onset Hypertensive Disorders of Pregnancy (per 1000 Live Births) in Each Maternal Age, Race and Ethnicity Group in the First (1951-1959) and Last (1996-2004) Cohort (Maternal Birth Year) Adjusted for Non-Linear Period (Calendar Year of Delivery) Effects

Age (years) -Race/Ethnicity Group	Cohort (Maternal Birth Year)	
	1951-1959	1996-2004
**Age 15-19 years**		
Hispanic	16.15 (12.08, 21.57)	55.11 (52.94, 57.38)
Non-Hispanic American Indian/Alaskan Native	43.87 (30.01, 64.12)	83.22 (78.54, 88.17)
Non-Hispanic Asian/Pacific Islander	14.00 (11.53, 17.00)	46.58 (41.21, 52.65)
Non-Hispanic Black	25.51 (21.31, 30.53)	83.14 (80.63, 85.73)
Non-Hispanic White	29.00 (26.03, 32.31)	73.33 (70.64, 76.11)
**Age 20-24 years**		
Hispanic	18.88 (14.14, 25.22)	64.46 (61.43, 67.64)
Non-Hispanic American Indian/Alaskan Native	53.84 (36.85, 78.65)	102.13 (95.54, 109.17)
Non-Hispanic Asian/Pacific Islander	15.10 (12.51, 18.23)	50.24 (43.76, 57.67)
Non-Hispanic Black	31.10 (26.00, 37.21)	101.38 (97.84, 105.03)
Non-Hispanic White	36.63 (32.90, 40.78)	92.62 (88.86, 96.55)

© 2022 Cameron NA et al. *JAMA Network Open.*

Age (years) -Race/Ethnicity Group	Cohort (Maternal Birth Year)	
**Age 25-29 years**	**1951-1959**	**1996-2004**
Hispanic	22.31 (16.70, 29.8)	76.14 (72.36, 80.12)
Non-Hispanic American Indian/Alaskan Native	62.67 (42.88, 91.59)	118.88 (110.66, 127.72)
Non-Hispanic Asian/Pacific Islander	15.23 (12.64, 18.36)	50.69 (44.17, 58.17)
Non-Hispanic Black	37.58 (31.41, 44.97)	122.5 (117.95, 127.21)
Non-Hispanic White	40.14 (36.06, 44.68)	101.49 (97.33, 105.83)
**Age 30-34 years**		
Hispanic	26.79 (20.05, 35.79)	91.44 (86.59, 96.57)
Non-Hispanic American Indian/Alaskan Native	74.38 (50.89, 108.72)	141.1 (130.34, 152.74)
Non-Hispanic Asian/Pacific Islander	19.76 (16.40, 23.81)	65.75 (57.23, 75.52)
Non-Hispanic Black	45.29 (37.84, 54.20)	147.62 (141.79, 153.69)
Non-Hispanic White	41.13 (36.94, 45.78)	103.99 (99.63, 108.53)
**Age 35-39 years**		
Hispanic	35.55 (26.59, 47.54)	121.36 (113.99, 129.21)
Non-Hispanic American Indian/Alaskan Native	96.19 (65.71, 140.8)	182.46 (165.45, 201.23)

© 2022 Cameron NA et al. *JAMA Network Open.*

Age (years) -Race/Ethnicity Group	Cohort (Maternal Birth Year)	
**Age 35-39 years**	**1951-1959**	**1996-2004**
Non-Hispanic Asian/Pacific Islander	31.49 (26.12, 37.96)	104.78 (90.99, 120.65)
Non-Hispanic Black	58.13 (48.55, 69.60)	189.48 (180.93, 198.42)
Non-Hispanic White	50.01 (44.91, 55.68)	126.44 (120.82, 132.33)
**Age 40-44 years**		
Hispanic	50.08 (37.95, 66.07)	170.94 (155.51, 187.89)
Non-Hispanic American Indian/Alaskan Native	141.51 (100.02, 200.22)	268.45 (228.12, 315.91)
Non-Hispanic Asian/Pacific Islander	48.45 (40.62, 57.79)	161.22 (138.76, 187.31)
Non-Hispanic Black	77.28 (65.28, 91.47)	251.88 (235.11, 269.84)
Non-Hispanic White	64.00 (58.03, 70.59)	161.83 (152.62, 171.61)

© 2022 Cameron NA et al. *JAMA Network Open.*

eFigure 1. Study Population Utilized From the National Vital Statistics System

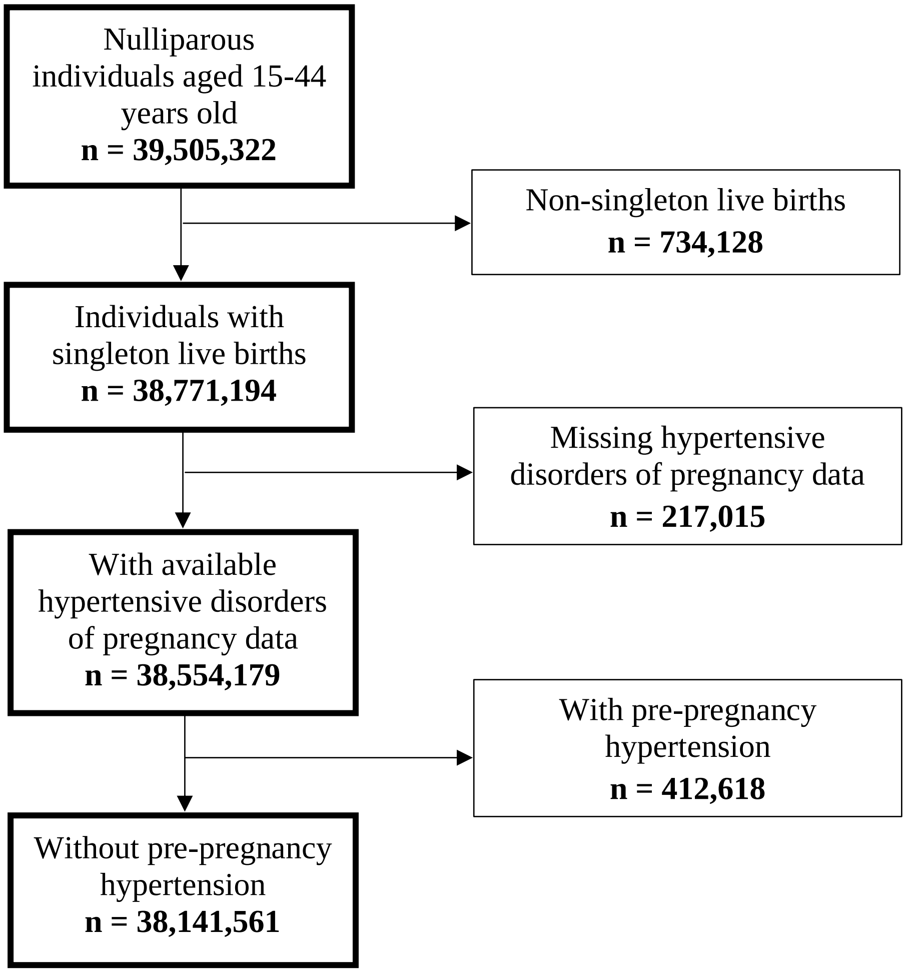

eFigure 1 Legend. The study population included nulliparous individuals with a singleton first live birth without hypertension prior to pregnancy and available hypertensive disorders of pregnancy data in the National Vital Statistics System

© 2022 Cameron NA et al. *JAMA Network Open.*

eFigure 2. Estimated Incidence of New-Onset Hypertensive Disorders of Pregnancy (per 1000 Live Births) in Each Maternal Age at Delivery, Race and Ethnicity in the First (1951-1959) and Last (1996-2004) Cohort (Maternal Birth Year) Adjusted for Non-Linear Period (Calendar Year of Delivery) Effects

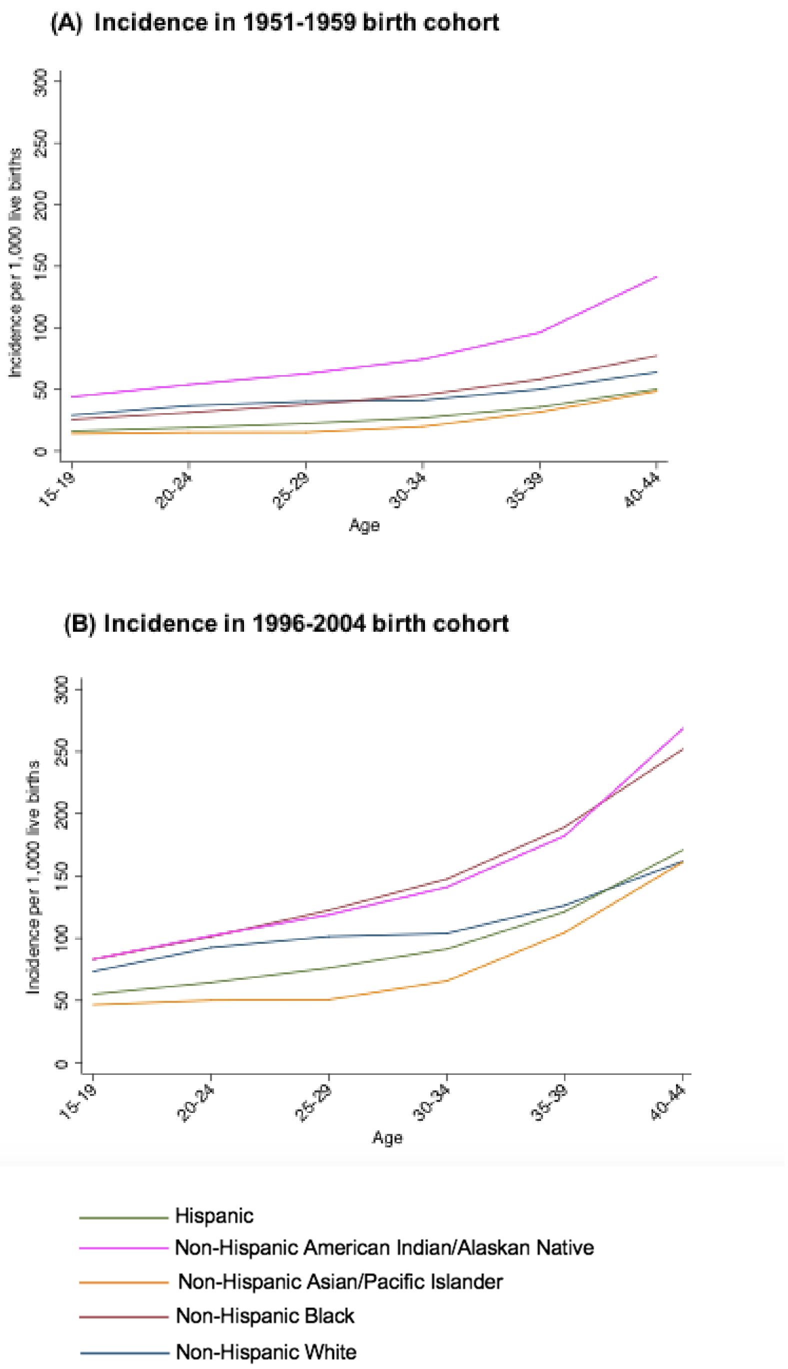

© 2022 Cameron NA et al. *JAMA Network Open.*

eFigure 2 Legend. Incidence of new-onset hypertensive disorders of per 1,000 live births were higher with older maternal age and more recent birth cohort in each racial and ethnic group after adjustment for non-linear period effects

© 2022 Cameron NA et al. *JAMA Network Open.*
